# World Encephalitis Day — February 22, 2014

**Published:** 2014-02-21

Encephalitis, inflammation of the brain, is caused by several different infectious and noninfectious entities. Encephalitis can be an uncommon complication of a common infection, such as infection with a herpes virus or with any of several vaccine-preventable disease viruses, or a predictable presentation of a rare pathogen, such as the ameba, *Naegleria fowleri*.

The epidemiology of encephalitis is influenced by many factors, including vaccine availability, global travel, and environmental alterations, such as climate change (1). Some encephalitis etiologies, such as the measles, mumps, rubella, and varicella viruses, have been virtually eliminated in certain settings through vaccination. However, other encephalitis pathogens have emerged or reemerged, including West Nile virus, Nipah virus, European tickborne encephalitis virus, enterovirus 71, and the ameba, *Balamuthia mandrillaris*.

Determining the cause of encephalitis can be difficult in part because of its nonspecific clinical presentation and the large number of causative agents. Despite exhaustive testing, an etiology is only identified in 40%–80% of cases (2). Moreover, autoimmune and infectious encephalitides are clinically indistinguishable (3).

The overall case-fatality rate of encephalitis is 5%–30%, but individual outcomes are highly dependent on the underlying cause and host factors, and survivors are often left permanently disabled. For instance, rabies is almost universally fatal, whereas encephalitis from enterovirus infection generally has better outcomes. Encephalitis also has substantial health-care costs given its severity as well as complexity of diagnosis and treatment (2).

Despite these challenges, progress is being made. World Encephalitis Day will be observed on February 22, 2014, to draw attention to encephalitis and invigorate global prevention efforts (4–6).
